# Supplementary material for: SCMarker: Ab initio marker selection for single cell transcriptome profiling
Source: PLoS Comput Biol. 2019 Oct 28;15(10):e1007445. doi: 10.1371/journal.pcbi.1007445 (PMC6837541; doi:10.1371/journal.pcbi.1007445)
Supplement: S1 Table — (DOCX) [file pcbi.1007445.s001.docx]

**S1 Table. Clustering methods.**

| **Method** | **Description** |
| --- | --- |
| Ascend | PCA dimension reduction and iterative hierarchical cluster |
| CIDR | PCA dimension reduction and hierarchical clustering |
| FlowSOM | PCA dimension reduction, then self-organizing maps and hierarchical consensus meta-clustering |
| PCAHC | PCA dimension reduction and hierarchical clustering |
| PCAKmeans | PCA dimension reduction and Kmeans |
| pcaReduce | PCA dimension reduction and iterative Kmeans |
| RtsneKmeans | t-SNE dimension reduction and Kmeans |
| SAFE | Ensemble clustering using SC3, Seurat, and tSNE+Kmeans |
| SC3 | PCA dimension reduction, then kmeans and different dimensions and hierarchical clustering on consensus |
| SC3svm | SC3 and SVM |
| Seurat | PCA dimension reduction and nearest neighbor graph clustering |
| TSCAN | PCA dimension reduction and model based clustering |
