# Supplementary figures and images for: SCMarker: Ab initio marker selection for single cell transcriptome profiling
Source: PLoS Comput Biol. 2019 Oct 28;15(10):e1007445. doi: 10.1371/journal.pcbi.1007445 (PMC6837541; doi:10.1371/journal.pcbi.1007445)

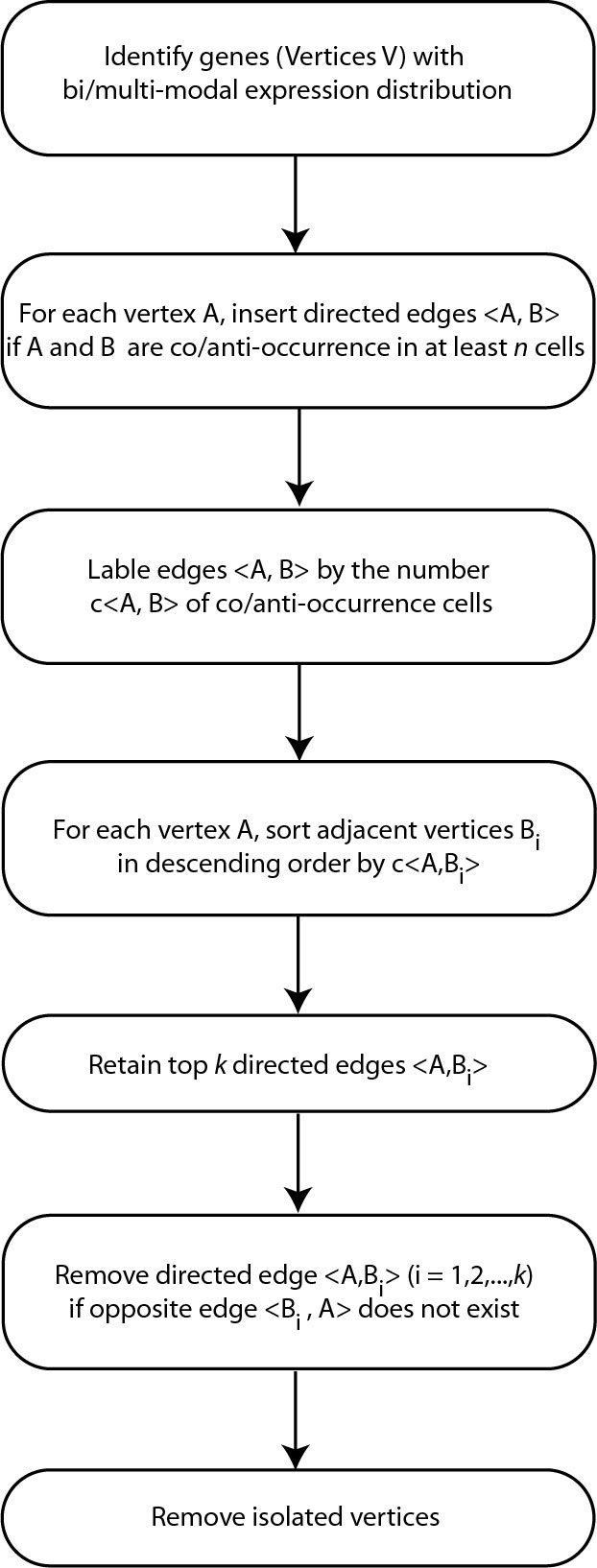

Supplement: S1 Fig — (JPG) [file pcbi.1007445.s003.jpg]

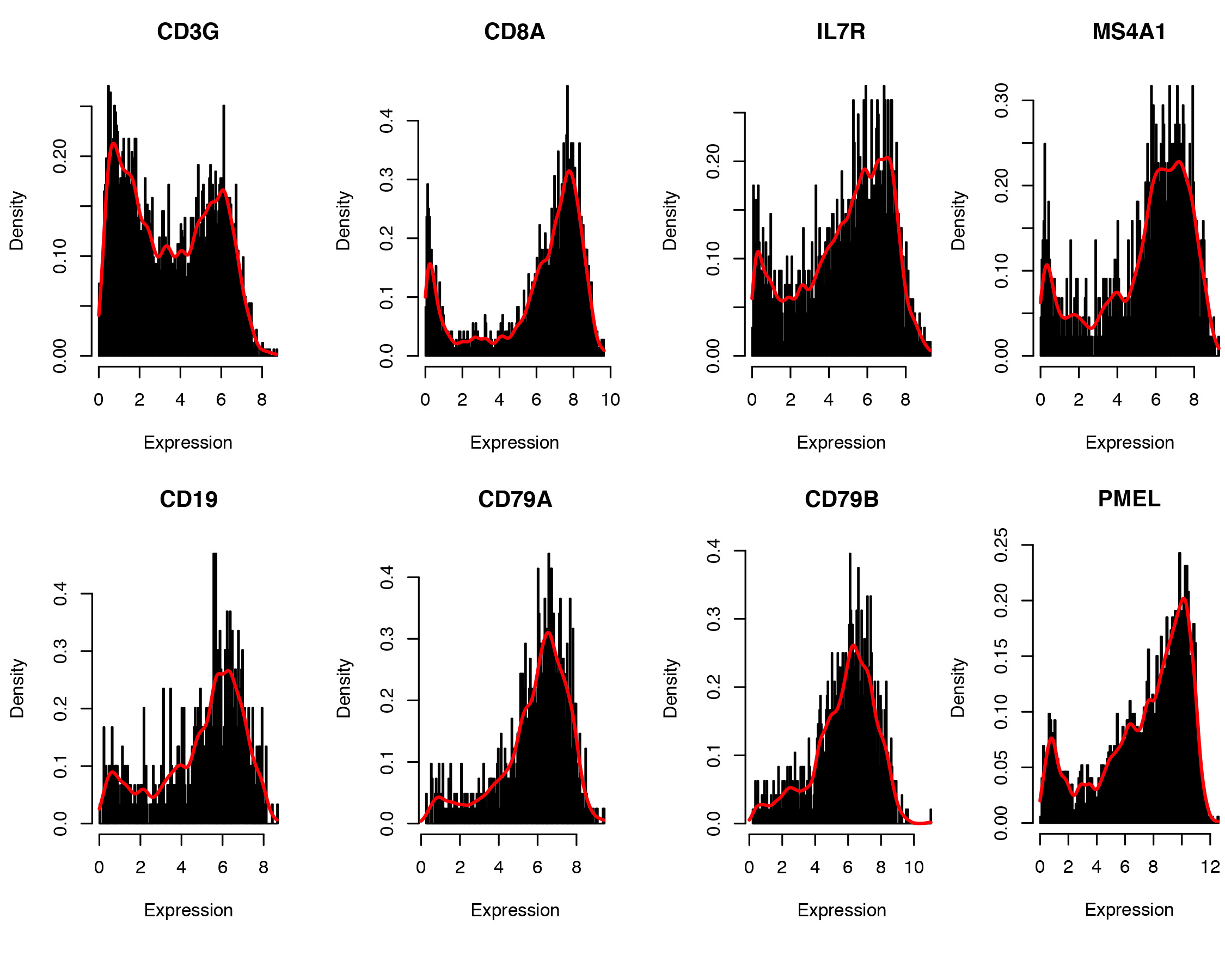

Supplement: S2 Fig — (JPG) [file pcbi.1007445.s004.jpg]

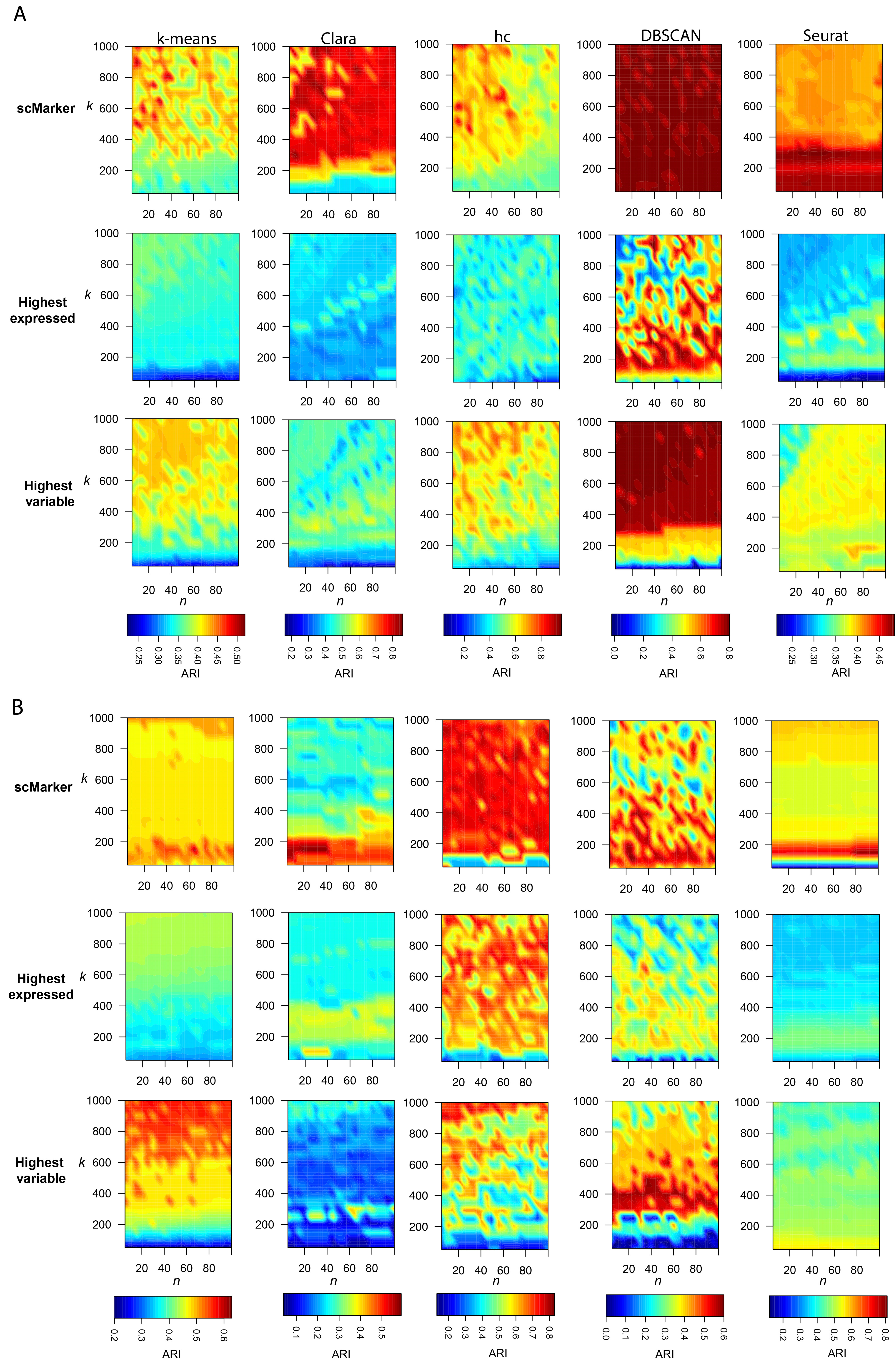

Supplement: S3 Fig — Tested were a range of parameters and 5 clustering algorithms: k-means, Clara, hierarchical clustering (hc), DBSCAN, and Seurat. Plotted in heatmaps are the ARI values calculated based on markers selected respectively by SCMarker, the highest expressed genes and the highest variable genes from (A) the melanoma and (B) the head and neck cancer data. X and Y axes in the SCMarker panel indicate the n and k parameters used by SCMarker and the corresponding (equal number of markers) results in the highest expressed or the highest variable gene panels. (JPG) [file pcbi.1007445.s005.jpg]

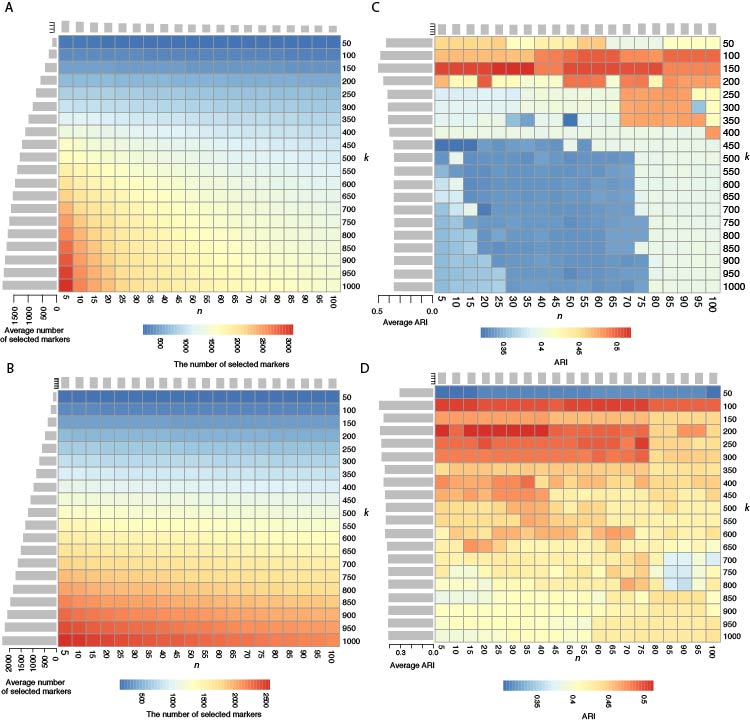

Supplement: S4 Fig — Plotted in the heatmaps are the number of selected markers for (A) the melanoma and (B) the head and neck cancer data over a range of n (X-axis) and k (Y-axis) parameters. Bars on the side and the top are the mean values in the corresponding rows and columns. Also plotted are clustering accuracy measured by the adjusted rand index (ARI), a metric that measures the similarity of two clustering results, for (C) the melanoma and (D) the head and neck cancer data over various n and k parameters. (JPG) [file pcbi.1007445.s006.jpg]

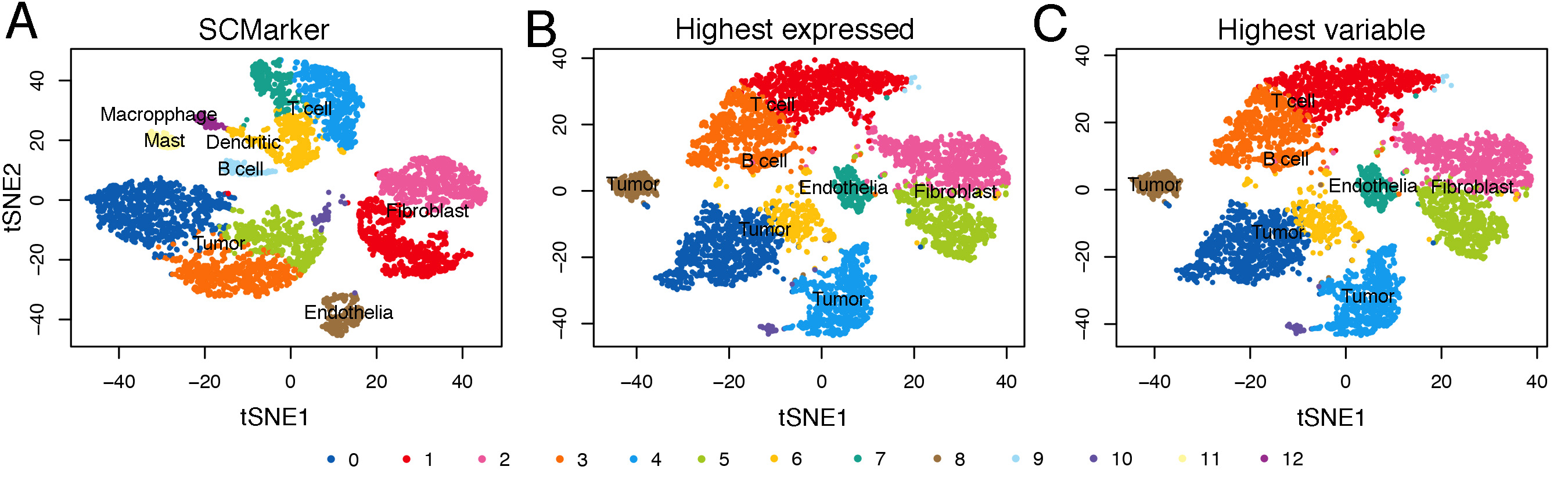

Supplement: S5 Fig — Plotted in tSNE space are 5,902 cells from the head and neck cancer data, based on genes selected respectively by (A) SCMarker, (B) the highest expressed and (C) the highest variable genes. (JPG) [file pcbi.1007445.s007.jpg]

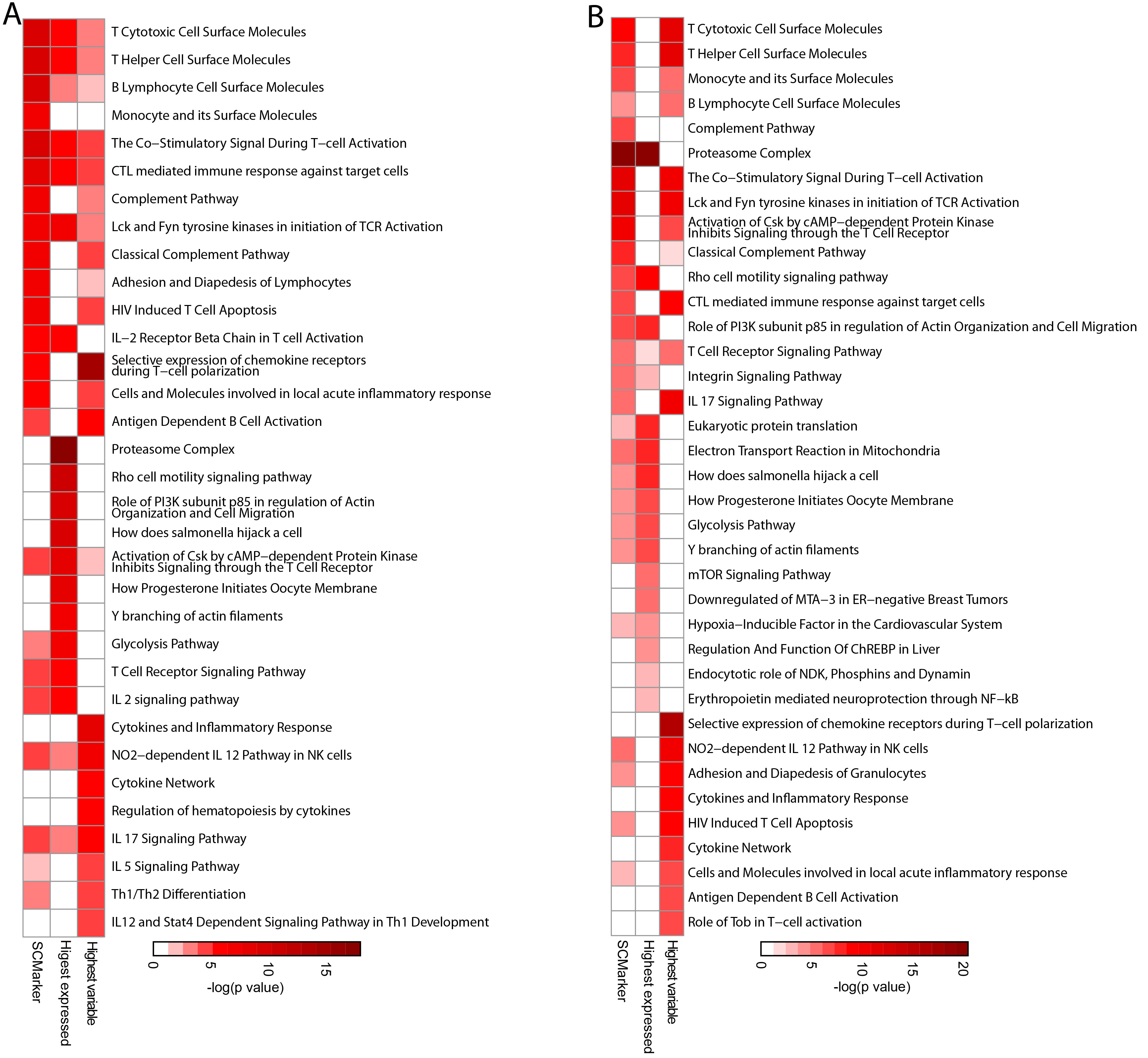

Supplement: S6 Fig — Gene set enrichment analysis (GSEA) of markers selected by 3 methods: SCMaker, the highest expressed and the highest variable genes from the (A) melanoma; and (B) the head and neck cancer data, respectively. Only the top 15 terms are shown. The darkness of the colors corresponds to -log10 P values. (JPG) [file pcbi.1007445.s008.jpg]

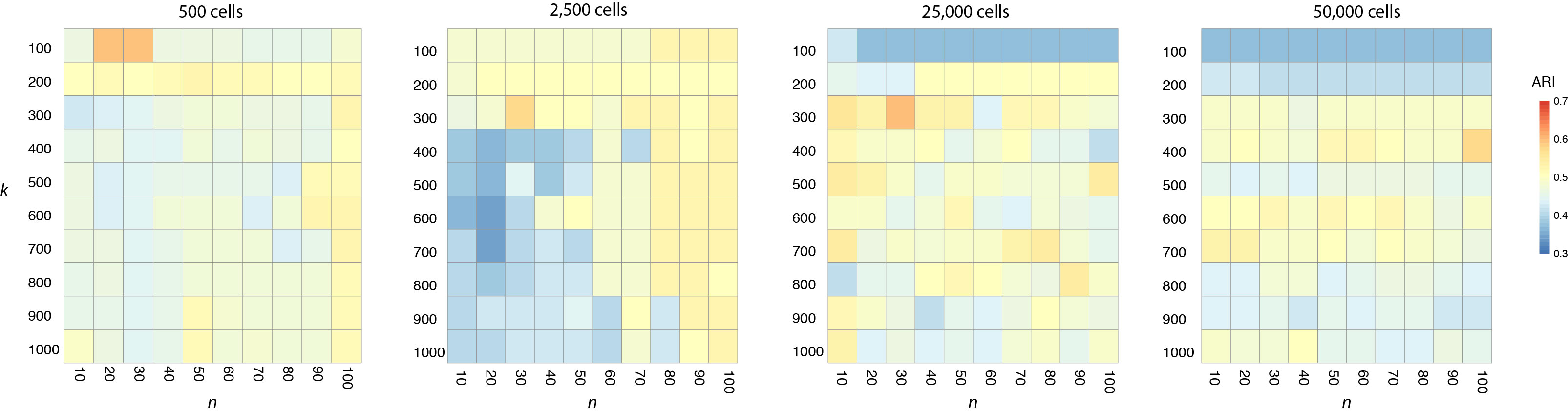

Supplement: S7 Fig — Plotted in heatmap are clustering accuracy measured by the adjusted rand index (ARI). The sample sizes of each dataset were labelled above each of the figures. (JPG) [file pcbi.1007445.s009.jpg]

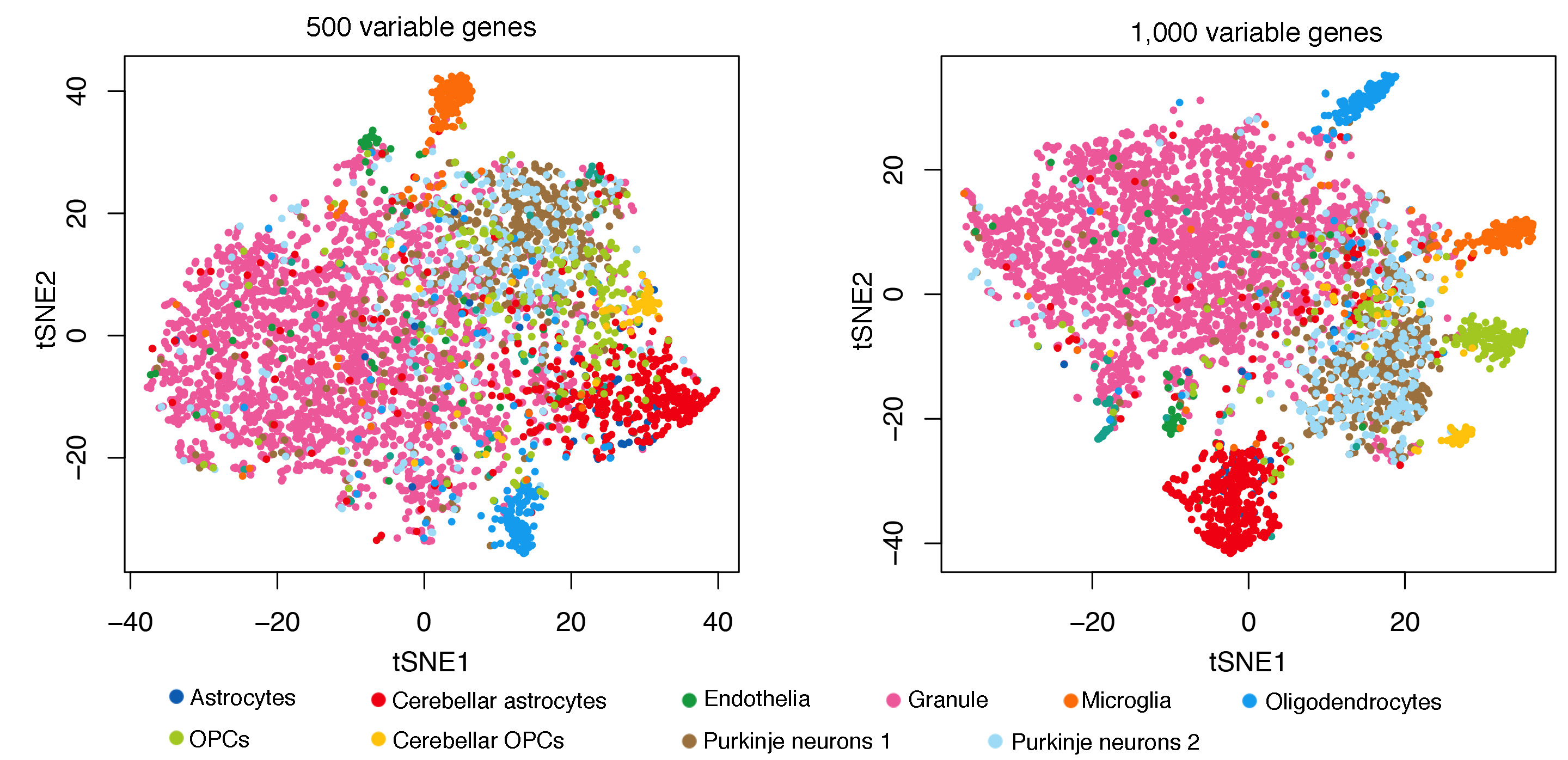

Supplement: S8 Fig — Plotted in tSNE space are 5,602 cells in the cerebellar hemisphere of human brain tissue based on the highest 500 (A) and 1000 (B) variable genes, colored by cell types from the original paper. (JPG) [file pcbi.1007445.s010.jpg]

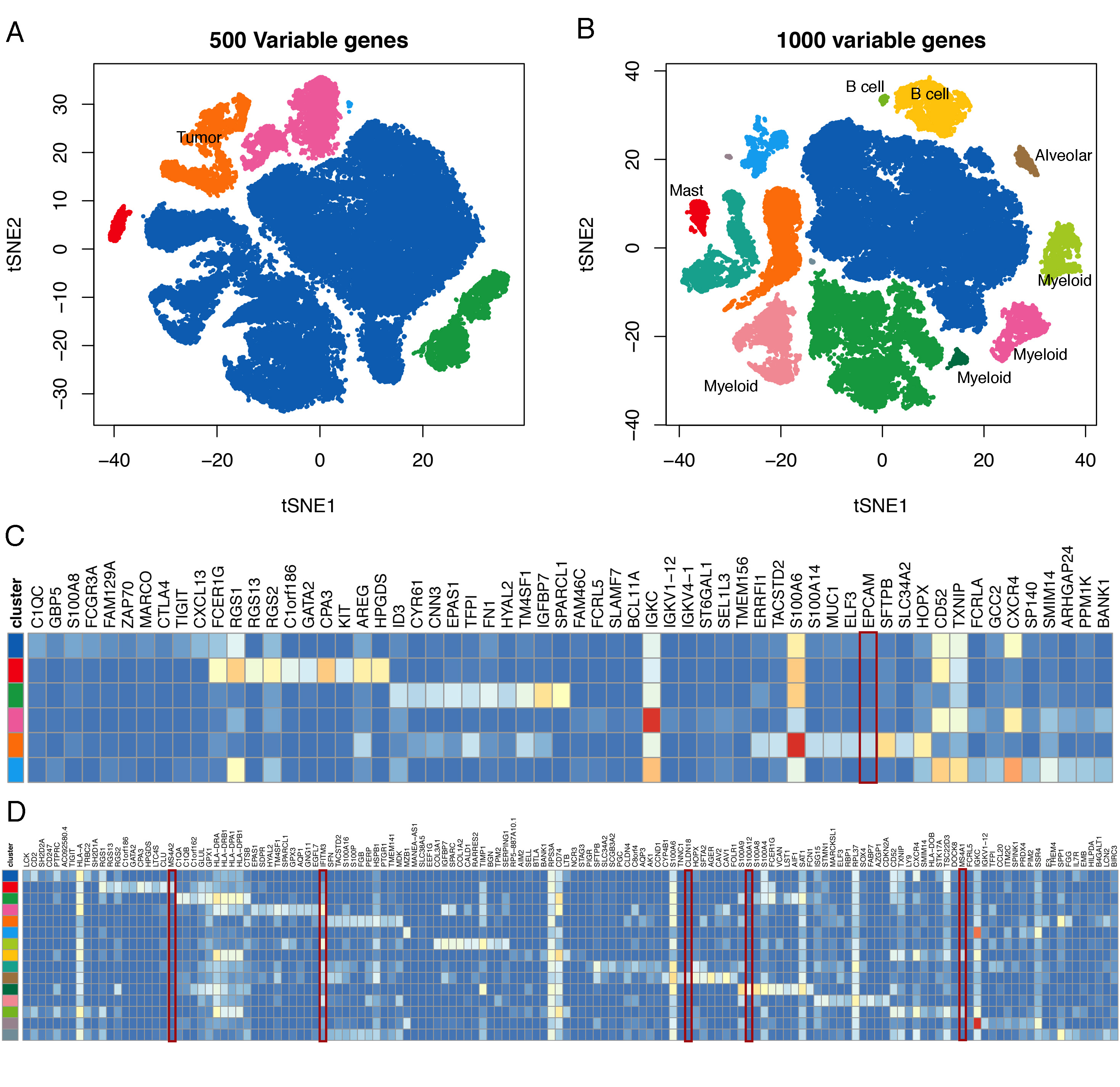

Supplement: S9 Fig — Plotted in tSNE space are 52,698 cells of 6 different lung cancer patients, clustered based on the highest 500 (A) and 1000 (B) variable genes. Colors correspond to clusters determined by DBSCAN. Heatmaps of the average expression levels of the 10 highest expressed genes per cluster identified respectively by the highest 500 (C) and 1000 (D) variable genes. Cell types in (A) and (B) are labelled based on the known cell-type specific markers, which are highlighted in red box in (C) and (D). (JPG) [file pcbi.1007445.s011.jpg]

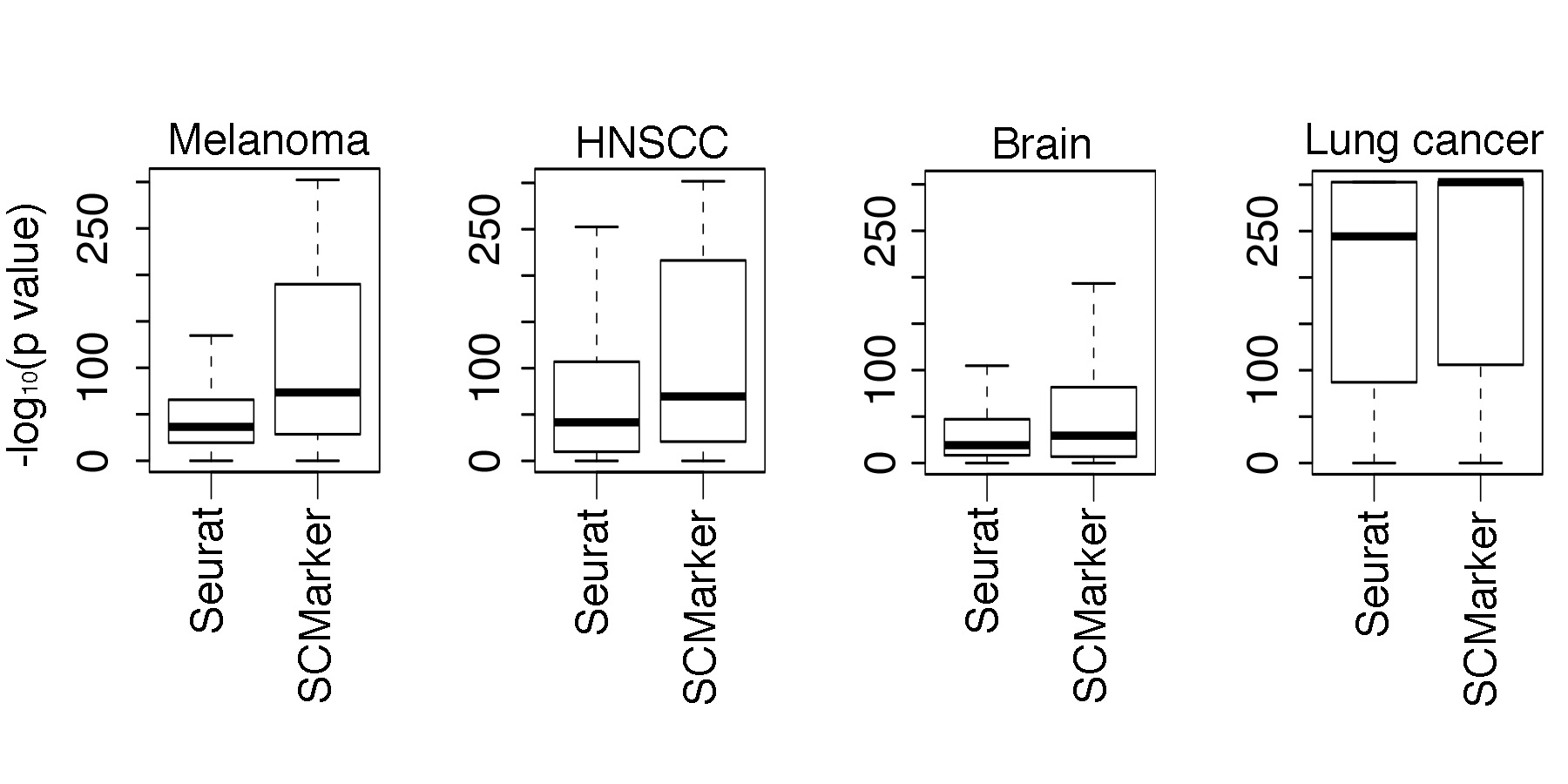

Supplement: S10 Fig — (JPG) [file pcbi.1007445.s012.jpg]
